# Protein Glycoengineering: An Approach for Improving Protein Properties

**DOI:** 10.3389/fchem.2020.00622

**Published:** 2020-07-23

**Authors:** Bo Ma, Xiaoyang Guan, Yaohao Li, Shiying Shang, Jing Li, Zhongping Tan

**Affiliations:** ^1^State Key Laboratory of Bioactive Substance and Function of Natural Medicines, Institute of Materia Medica, Chinese Academy of Medical Sciences and Peking Union Medical College, Beijing, China; ^2^Department of Chemistry and Biochemistry and BioFrontiers Institute, University of Colorado, Boulder, CO, United States; ^3^School of Pharmaceutical Sciences, Tsinghua University, Beijing, China; ^4^Beijing Key Laboratory of DNA Damage Response and College of Life Sciences, Capital Normal University, Beijing, China

**Keywords:** glycoengineering, therapeutic protein, enzyme, biological method, chemical method

## Abstract

Natural proteins are an important source of therapeutic agents and industrial enzymes. While many of them have the potential to be used as highly effective medical treatments for a wide range of diseases or as catalysts for conversion of a range of molecules into important product types required by modern society, problems associated with poor biophysical and biological properties have limited their applications. Engineering proteins with reduced side-effects and/or improved biophysical and biological properties is therefore of great importance. As a common protein modification, glycosylation has the capacity to greatly influence these properties. Over the past three decades, research from many disciplines has established the importance of glycoengineering in overcoming the limitations of proteins. In this review, we will summarize the methods that have been used to glycoengineer proteins and briefly discuss some representative examples of these methods, with the goal of providing a general overview of this research area.

## Introduction

With the deepening of our understanding of biology, recombinant proteins have become an important class of biological macromolecules that are widely used in medicine, industry, agriculture, environmental protection, and other fields (Puetz and Wurm, 2019). In the arena of medicine, therapeutic proteins, such as antibodies, cytokines/growth factors, and hormones, are indispensable for the prevention and treatment of cancer, infections, autoimmune diseases, metabolic genetic diseases, and many other diseases, largely due to their advantages of high specificity, low toxicity, and defined biological functions. They are now the fastest-growing segment of the global pharma market (Owczarek et al., 2019). Proteins that are frequently utilized in industrial, agricultural, environmental protection, and other related fields are enzymes, which include amylase, lactase, lipase, phytase, xylanase, and cellulase. Enzymes have the advantages of high catalytic efficiency, high specificity, mild reaction conditions, and less pollution. Their applications in food, detergent, textile, paper, breeding, new energy, and waste management industries have greatly improved the quality of produced products, reduced environmental pollution, and promoted sustainable economic and ecological development (Arbige et al., 2019).

However, due to the nature of biological macromolecules, proteins also have their own problems. Because of their large molecular weight, complex composition and structure, many proteins have limited solubility and thermal and proteolytic stability. They can be denatured during storage or are prone to aggregation and chemical modifications, such as oxidation and deamidation. The existence of these problems can result in decreased efficacy of therapeutic proteins and increased immunogenic side effects. For enzymes, these problems could lead to their slow development and high production costs, which in turn limit their industrial applications. Scientists have been trying for many years to solve these problems (Sinha and Shukla, 2019). They have explored many different methods to engineer proteins, with the hope of improving their stability, solubility, and biological activity, decreasing the immunogenicity or other side effects of therapeutic proteins, and reducing the production costs of industrial enzymes. Among all the methods tested, glycoengineering appeared to be one of the most promising for future research.

**Figure 1:**
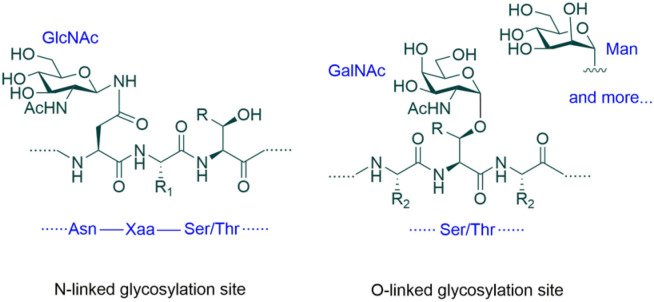
Protein glycosylation. The two most common types of glycosylation are N- and O-linked glycosylation. The consensus sequence for N-glycosylation is Asn-Xaa-Ser/Thr (where Xaa is not Pro). No consensus sequence for O-glycosylation has been established. In eukaryotic cells, the first sugar residue of N-glycans is usually β-GlcNAc, while the first residue of O-glycans can be β-GlcNAc, α-GalNAc, α-Man or other monosaccharide units. R = H or Me, R_1_, and R_2_ = Amino acid side chains.

Glycoengineering is a method of improving the properties of proteins by changing their glycosylation (Goochee et al., 1991; Sinclair and Elliott, 2005; Beck and Reichert, 2012; Dicker and Strasser, 2015). Glycosylation of proteins refers to the attachment of glycans to proteins in the form of covalent bonds (Figure 1) (Spiro, 2002). Glycans can also be called carbohydrates, sugars, monosaccharides, oligosaccharides, or polysaccharides. Glycosylation is a major form of post-translational modification (PTM) of proteins. Glycosylation can occur on the side chains of many amino acid residues of proteins in a number of different ways. The two most common ways are to attach glycans to the side chain nitrogen (N) atoms of Asn residues and to the side chain oxygen (O) atoms of Ser and Thr residues. Depending on atoms to which glycans are linked, these two types of glycosylation are called N-linked glycosylation and O-linked glycosylation, respectively. In addition to the different side chain atoms in the glycosidic linkage, there are also many other differences between these two types of glycosylation. For example, in eukaryotic cells where glycosylation is widely present, the first sugar residue that is directly attached to Asn is usually β-linked N-acetylglucosamine (β-GlcNAc), while the ones on Ser and Thr side chains include many different structures, such as β-GlcNAc, α-linked N-acetylgalactose (α-GalNAc), α-linked mannose (α-Man), α-linked fucose, β-linked xylose, α- or β-linked galactose and glucose (Figure 1).

Protein with glycosylation are called glycoproteins. Many years of research has demonstrated that glycosylation is an important PTM that plays important roles in regulating the properties of proteins (Rudd et al., 1994; Boyd et al., 1995; Van den Steen et al., 1998). By forming hydrogen bonds or other non-covalent interactions with amino acid residues of the proteins to which they are attached, glycans can improve the folding efficiency and conformational stability of proteins, prevent their abnormal aggregation, increase their water solubility, and decrease their rate of thermal denaturation, proteolytic inactivation and chemical degradation (Varki, 2017). In addition, glycans can also directly participate in the interaction with other macromolecules, viruses, and cells, thereby leading to altered substrate binding affinity and specificity, and biological activity of glycoproteins. Compared with other types of PTMs and amino acid mutations, the greatest advantage of glycoengineering is that, when glycosylation sites and glycan structures are selected appropriately, this method is capable of simultaneously improving many different properties of proteins. Such an advantage has aroused great interest of scientists to explore this new frontier.

Since the 1980s, scientists have started to use glycosyltransferases and glycosidases to add sugars to and remove sugars from oligosaccharide chains of proteins by utilizing *in vivo* (cellular) genetic technologies and *in vitro* enzymatic methods (Lee et al., 1989; Lairson et al., 2008; Bennett et al., 2012; Albesa-Jove et al., 2014; Janetzko and Walker, 2014; Moremen and Haltiwanger, 2019). Their efforts have led to many important findings, and the discovery and development of many therapeutic proteins and enzymes with improved properties and functions. But on the whole, the number of successfully commercialized enzymes and approved therapeutic proteins that have been developed through protein glycoengineering is small, with probably the most well-known one being darbepoetin alfa, a novel therapeutic agent for renal anemia (Elliott et al., 2003). A possible explanation for the small number is that sufficient understanding of the structure-function relationship of protein glycosylation has not been achieved and reliable scientific theories have not been fully developed to guide the glycoengineering efforts. In order to improve the success rate of protein glycoengineering, scientists need to conduct more research into the relationship between the structure and performance of glycoproteins. Although it may take a long time to establish reliable guidelines for predicting the outcomes of protein glycoengineering, more and more encouraging results have been obtained in recent years. In this review, we will summarize and compare some of the representative results, with the goal of providing a general picture of this research area.

This review is intended to provide a brief introduction to the protein glycoengineering area. We will only touch upon a limited number of examples for each research direction. Interested readers may refer to more comprehensive reviews for detailed information (Bailey, 1991; Wright and Morrison, 1997; Saxon and Bertozzi, 2001; Bretthauer, 2003; Sinclair and Elliott, 2005; Hamilton and Gerngross, 2007; Beck et al., 2008; Beck and Reichert, 2012; Beckham et al., 2012; Baker et al., 2013; Merritt et al., 2013; Dicker and Strasser, 2015; Geisler et al., 2015; Greene et al., 2015; Buettner et al., 2018; Mimura et al., 2018; Montero-Morales and Steinkellner, 2018; Tejwani et al., 2018; Wang et al., 2018, 2019; Yates et al., 2018; Agatemor et al., 2019; Harding and Feldman, 2019; Mastrangeli et al., 2019). In addition to glycoengineering using naturally occurring glycans and glycosidic linkages to improve the properties of proteins, there are many research efforts geared toward chemical and enzymatic synthesis of glycans, development of glycan-based vaccines and adjuvants, or using unnatural glycans and site-selective conjugation chemistry to achieve protein glycoengineering objectives. Detailed discussions of these efforts are beyond the scope of this review. The necessary information about these research studies can be found in excellent review articles by Saxon and Bertozzi (2001), Sola et al. (2007), Gamblin et al. (2009), Wolfert and Boons (2013), Krasnova and Wong (2016), Wu et al. (2017), Sun et al. (2018), Wen et al. (2018), Guberman and Seeberger (2019), Moremen and Haltiwanger (2019), and Rahfeld and Withers (2020).

Protein glycosylation is defined by glycosylation sites and glycan structures. Accordingly, protein glycoengineering is carried out by varying two parameters: site and structure, and more specifically, by changing the number and position of the glycosylation sites and/or by changing the structure of glycans (including linkage type, chain length, and composition) at individual glycosylation sites. Based on the way how glycoproteins are produced, protein glycoengineering can be roughly divided into two main categories (Wang et al., 2019). In one category, glycoproteins are produced by cell expression. In the other category, they are prepared through chemical synthesis, including biochemical and organic synthesis (Rich and Withers, 2009). Here, we will first review glycoengineering methods based on cell expression, and then discuss chemical synthesis-based glycoengineering methods.

## Cell-Based Protein Glycoengineering

In the past 30 years, many different methods have been developed to engineer cells of animals, plants, insects, yeasts, bacteria, etc. to express proteins with desired glycosylation patterns. These methods mainly use gene knockout, knockdown, knock-in, overexpression, mutation, or small molecule suppression technologies to change the type and concentration of glycosidases and glycosyltransferases that are available inside these cells, thereby changing the glycosylation patterns of interested proteins expressed in them. Recent advances in gene editing tools, especially the CRISPR/Cas9 system, has enabled more rapid and cost-effective cell glycoengineering (Chan et al., 2016; Chung et al., 2017; Mabashi-Asazuma and Jarvis, 2017; Jansing et al., 2019; Karottki et al., 2020). Currently, the most widely used cells for protein glycoengineering are mammalian cells.

### Glycoengineering Based on Mammalian Cells

Since the 1980s, mammalian cells, mainly Chinese hamster ovary (CHO) cells, have been used for the production of glycosylated recombinant therapeutic proteins (Tejwani et al., 2018; Wang et al., 2018). Compared to human cell lines, CHO cells tend to add a small amount of non-human glycans α-galactose (α-Gal) and N-glycolylneuraminic acid (Neu5Gc) to recombinant proteins (Hokke et al., 1990). If their quality is not well controlled, engineered glycoproteins produced by this expression system may cause immune response. Despite this minor limitation, CHO cells offer multiple advantages. First, they can be cultured in large-scale bioreactors and their production rate of glycoproteins is much higher than that of human cells. Second, due to the natural differences in species, CHO cells are much less likely to transmit human pathogens. Because the advantages outweigh the disadvantages, CHO cells have become one of the most widely used mammalian cell expression system for the production of glycoproteins.

**Figure 2:**
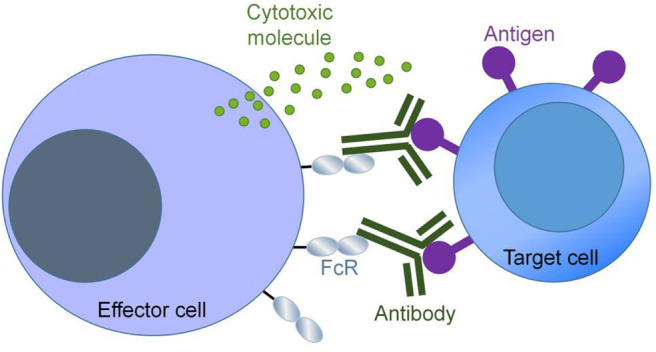
Antibody-dependent cellular cytotoxicity (ADCC). ADCC is triggered when the Fab domain of an antibody binds to an antigen on the target cell and the Fc domain of the same antibody molecule binds to the FcR on the effector cell.

One protein glycoengineering strategy based on CHO cells is to modify the structure of glycans on proteins through gene knockout technologies, so as to achieve the goal of improving their properties. A representative work in this regard is to enhance the antibody-dependent cell-mediated cytotoxicity (ADCC) of immunoglobulin (IgG) antibodies by knocking out α-1,6-fucosyltransferase (FUT8). ADCC is an important mechanism of antibody therapeutics. Antibodies recognize and bind to surface antigens of target cells (e.g., cancer cells) through the antigen binding fragments (Fab), and interact with crystalline fragment (Fc) receptors (FcR) on effector cells (such as natural killer cells) via the Fc portion. After the interaction of Fc with FcR, immune effector cells are activated and secrete cytotoxic molecules to kill target cells. This process is called ADCC (Figure 2). Enhancement of ADCC can be achieved by increasing the binding affinity of antibodies to Fc receptors, which in turn can be accomplished by modifying the glycosylation of the Fc region of IgG.

The highly conserved Asn residues at position 297 (N297) of the IgG Fc regions are N-glycosylated (Wright and Morrison, 1997; Beck et al., 2008; Reusch and Tejada, 2015; Mastrangeli et al., 2019). Previous studies have found that the fucose residue attached via α-1,6-linkage to the innermost N-GlcNAc of the N-glycans at N297 is the key residue for modulating ADCC. Removal of the core fucose moiety from IgG-Fc glycans can significantly increase the binding affinity of Fc for FcR, thereby enhancing ADCC (Shields et al., 2002). FUT8 is the sole enzyme that catalyzes the transfer of fucose from GDP-fucose to N-linked oligosaccharides. Therefore, knocking out the *FUT8* gene in CHO cells would be a promising method for producing therapeutic IgG antibodies with enhanced ADCC (Yamane-Ohnuki et al., 2004). This concept was validated experimentally. In a representative study, Yamane-Ohnuk et al. successfully generated *FUT8*^−^^/−^ CHO/DG44 cell lines by sequential homologous recombination. Their expression results showed that the anti-CD20 (IgG1) antibody produced by their cell line had significantly increased binding affinity to the human receptor FcγRIIIa, and the ADCC of this antibody was enhanced to ~ 100-fold compared with that produced in normal CHO/DG44 cells.

**Figure 3:**
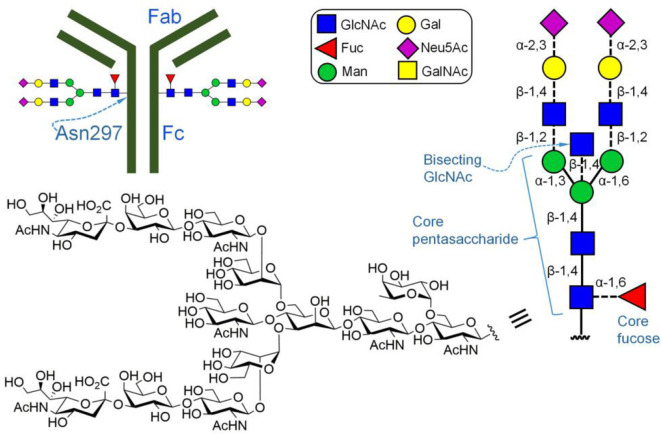
Fc-glycosylated immunoglobulin G (IgG). Depicted is a complex type N-linked glycan with a core fucose.

Previous studies have shown that other monosaccharides of N-glycans attached to Asn297 of IgG could also regulate ADCC (for example, the bisecting GlcNAc linked β-1,4 to the mannosyl residue in the core pentasaccharide Figure 3; Davies et al., 2001). During the biosynthesis of N-glycans, the key enzyme that catalyzes the introduction of bisecting GlcNAc into N-glycans is the β-1,4-N-acetylglucosaminyltransferase III (GnTIII). Based on this knowledge, Umana et al. (1999) constructed a GnTIII cDNA transfected CHO cell line. By promoting the overexpression of GnTIII, they were able to obtain IgG antibodies with increased bisecting GlcNAc. Their results showed that the ADCC of the produced IgG antibody is much higher than that of antibodies containing less bisecting glycans, suggesting that bisecting GlcNAc has a positive impact on ADCC.

However, although both fucose removal and bisecting GlcNAc addition enhance ADCC of antibodies, the magnitude of the increase caused by these two different types of modifications is quite different. The increase caused by the modification of N-glycans by bisecting GlcNAc is generally less than 10-fold, which is much lower than that observed with the removal of fucose. In addition, the success rates of these two methods are also different. Glycoengineering carried out by removing fucose residues has a higher success rate than that by adding bisecting GlcNAc. Indeed, Yamane-Ohnuki et al. (2004) has argued that bisecting GlcNAc may have no effect on ADCC. Therefore, the former method is currently more widely used.

The high variability and controversial reliability of the results of protein glycoengineering based on bisecting GlcNAc was related to the previous lack of a clear and definite understanding of this type of glycosylation and how it is regulated (Shinkawa et al., 2003). These glycoengineering studies were performed using empirical knowledge. Without a theoretical foundation, little was known about how glycosylation affects protein properties, and under what circumstances it could improves protein properties. The glycoengineering design in such a way is not very scientific and therefore would inevitably produce controversial results. To reverse this situation, a deeper and clearer understanding of protein glycosylation is required. An excellent example demonstrating this point is the work by Ferrara et al. (2006). Through their research, they found that the high bisecting GlcNAc level introduced by overexpression of GnTIII inhibited the core fucosylation, which led to an increase in the proportion of N-glycans without fucose (Ferrara et al., 2006). This finding suggested that the bisecting GlcNAc may regulate ADCC indirectly and therefore, its effect is not very predictable.

Increasing the content of other monosaccharides on N-glycans in the Fc region of IgG antibodies, such as the penultimate Gal and terminal N-acetylneuraminic acid (Neu5Ac/sialic acid) residues, has also been shown to improve the performance of antibodies, including enhancing their ADCC, complement-dependent cytotoxicity (CDC), and anti-inflammatory activities (Tsuchiya et al., 1989; Raymond et al., 2015). Similar to the previous uncertain role of bisecting GlcNAc in the ADCC, the effects of the presence of Gal and Neu5Ac on IgG antibodies are also not quite clear. Again, this is mainly due to the current lack of a deep understanding of protein glycosylation. The reason why it is difficult to improve the understanding of glycosylation is that there are not many available tools to accurately control or determine the composition of glycoproteins. For example, when increasing or decreasing the expression of one or more enzymes involved in the biosynthesis of glycans, it is hard to find a robust analytical tool that would allow one to assess whether the change in their expression would affect the functions of other glycosyltransferases and/or glycosidases. Even if this is not so, the inherent heterogeneity in the sugar moieties makes it difficult to describe precisely the composition of glycoproteins produced by recombinant host cells (Kodama et al., 1991; Higel et al., 2016). Glycosylation is not template-driven and heterogeneity of glycoproteins arises from the presence of different glycan structures at one glycosylation site (microheterogeneity) and different degrees of glycosylation site occupancy (macroheterogeneity). Due to the heterogeneity, glycoproteins typically exist as complex mixtures, which can consist of several tens to more than one hundred different glycoforms (Toll et al., 2006; Yang et al., 2016). The extent of heterogeneity can vary depending on glycoproteins and their production methods. Researchers from many different disciplines have undertaken considerable efforts to develop and optimize methods and tools for the control and analysis of the heterogeneity of recombinant glycoproteins and have achieved encouraging success. For example, by developing computation models of protein glycosylation, researchers are now able to provide guidance on the design of optimal strategies to obtain a target glycosylation profile with desired properties (Umana and Bailey, 1997; Grainger and James, 2013; Spahn et al., 2016, 2017; Krambeck et al., 2017; Sokolov et al., 2018; Liang et al., 2020). By improving chromatographic separation and analytical tools such as capillary electrophoresis, high performance liquid chromatography and mass spectrometry, researchers have made significant advances in the determination of the identity and quantity of differently glycosylated protein forms (glycoforms) (Domann et al., 2007; Zaia, 2008; Artemenko et al., 2012; Campbell et al., 2014; Zhang et al., 2016). Continued progress in these areas is expected to further broaden and deepen the understanding of the role of different monosaccharide units in regulating the properties of antibodies, thus making the cell-based glycoengineering results more predictable in the future.

**Figure 4:**
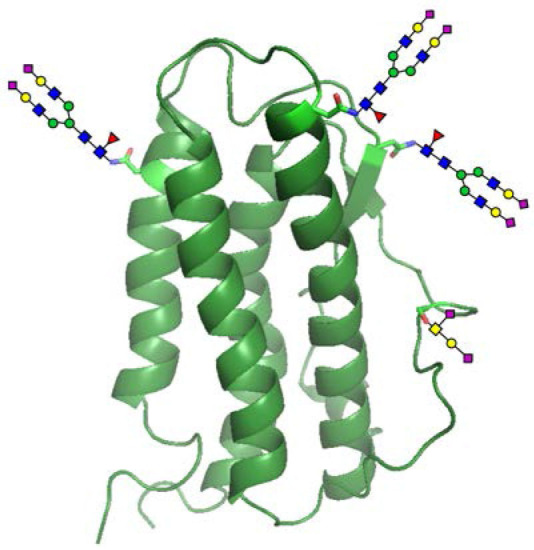
Glycosylated human erythropoietin (hEPO). Natural hEPO contains four glycosylation sites that are located at Asn24, Asn38, Asn83, and Ser126.

Besides changing glycan structure at specific glycosylation sites, glycoengineering can also be performed by changing the number of glycosylation sites. The most representative example in this regard is the glycoengineering of human erythropoietin (hEPO) (Egrie and Browne, 2001). The main medical use of hEPO is to treat anemia, especially anemia caused by chronic kindney disease, cancer radiotherapy and chemotherapy. The purpose of hEPO glycoengineering, simply put, is to extend its half-life *in vivo* by increasing the number of its N-linked glycosylation sites. Naturally occurring hEPO contains three N-glycosylation sites and one O-glycosylation site (Figure 4). Neu5Ac located at the terminal position of N-linked glycans is important for the circulating half-life of proteins because it can help reduce the chance of a protein being taken up into hepatocytes by endocytosis, filtered by the glomeruli, and degraded by proteases (Morell et al., 1971). Through careful research and analysis, Elliott et al. (2004) found that it is much easier to add new N-glycosylation sites to hEPO than to increase the number of O-linked ones. The main reason for this observation is that N-glycosylation sites are defined by the consensus sequence (or sequon), Asn-Xaa-Thr/Ser, where Asn is the glycosylation site and Xaa is any natural amino acid except Pro. Although it is not guaranteed that Asn residues in all consensus sequences can be glycosylated, the probability of them bearing N-glycans is very high. Unlike N-glycosylation, they found that O-glycosylation does not appear to be controlled by the primary sequence context and has no clear consensus sequences, and thought that it may be directed by the secondary or tertiary structure and occurs only in a very few sites that could meet its conformational requirements (Elliott et al., 1994). Guided by these empirical findings, Elliott et al. decided to only introduce new N-glycosylation sites into hEPO via site-directed mutagenesis. When the DNA sequence encoding the mutant hEPO was expressed in CHO cells, five N-glycans and one O-glycan were added to its surface. These two additional N-glycans greatly increased the content of Neu5Ac on hEPO, and thus helped reduce its rate of clearance from the bloodstream and improved its clinical efficacy.

In addition to the glycoengineering method of adjusting the expression and activity of enzymes involved in glycan biosynthesis and the method of increasing the number of glycosylation sites, another commonly used method is metabolic glycoengineering, a technique that was developed almost thirty years ago where protein glycosylation can be altered by changing the concentrations of monosaccharides or nucleotide sugars in the culture media (Bailey, 1991; Gramer et al., 2011; Buettner et al., 2018; Agatemor et al., 2019). A representative example is the study by Gu and Wang (1998) in which 20 mM of N-acetylaminomannose (ManNAc) was added to the culture media of CHO cells. They found that the supplement was able to decrease the proportion of incompletely sialylated N-glycans at Asn97 of interferon-γ (IFN-γ) from 35 to 20% without any adverse effect on cell growth and protein production. In mammalian systems, ManNAc is a metabolic precursor for the biosynthesis of Neu5Ac. It is converted into Neu5Ac in the cytosol, and following that, Neu5Ac enters the nucleus and is activated to form CMP-Neu5Ac. Finally, CMP-Neu5Ac is transported to the Golgi apparatus where Neu5Ac is transferred to an oligosaccharide chain. In this manner, the increase in the concentration of ManNAc leads to an elevated level of Neu5Ac, which in turn leads to an extended half-life of glycoproteins. In addition to ManNAc, a wide range of metabolite precursors, glycosyltransferase inhibitors, pH modulators, as well as cell culture parameters (e.g., pH, temperature) have also been explored for protein glycoengineering (Sha et al., 2016; Ehret et al., 2019). The glycoengineering method based on metabolism and based on the regulation of enzyme expression and activity are similar in principle, both of which achieve changes in glycan structures by interfering the pathway of N-glycan biosynthesis. It is thus conceivable that the metabolic glycoengineering method is also limited by the nature of the CHO cell expression system. Proteins glycoengineered using this method also exist as inseparable heterogeneous mixtures of glycoforms.

Apart from CHO cells, there are many other mammalian cell lines that have been utilized for protein glycoengineering, with the more frequent ones being mouse myeloma cells NS0 and SP2/0 (Lifely et al., 1995). The advantages of these cells for glycoengineering are very similar to those of CHO cells, i.e., they are also relatively easy to use and can give a high yield of proteins. Their disadvantages are also similar to those of CHO cells, that is, the engineered glycoproteins produced by the cells are in the form of heterogeneous mixtures, and may contain traces of non-human monosaccharides like α-Gal and Neu5Gc, etc.

### Glycoengineering Based on Non-mammalian Cells

**Figure 5:**
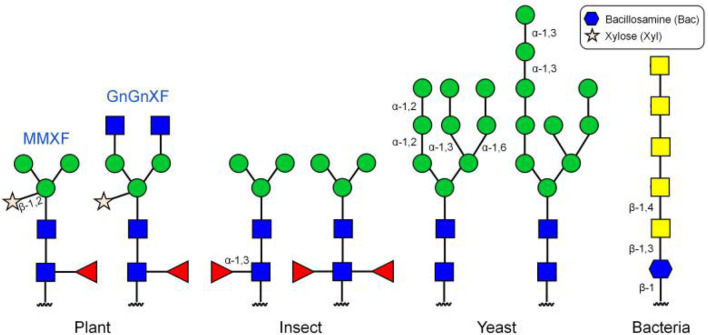
N-linked glycans on glycoproteins produced in different expression systems.

Scientists have also chosen many different types of non-mammalian cells for protein glycoengineering, including plant, insect, yeast, and bacteria cells. Compared with mammalian cells, plant cells have several advantages, the most important of which is that the glycoproteins produced in plant cells are more homogeneous than those synthesized in mammalian cells (Montero-Morales and Steinkellner, 2018). The reason for this is that plant cells normally produce only a few N-glycans, with two of them, namely GnGnXF and MMXF, accounting for more than 90% of the total (Figure 5) (Chen, 2016). Therefore, plant cells have the potential to generate glycoproteins with better defined N-glycan structures. A high degree of homogeneity would better help establish the detailed contribution of glycans to the physicochemical and biological properties of proteins and such information would be beneficial for protein glycoengineering. Other advantages of plant cells as a production host include fast production of glycoproteins and high tolerance toward manipulation of N-glycan biosynthetic pathways. A disadvantage of plant cells is that glycoproteins produced by such cells usually contain plant-specific core α-1,3-fucose and β-1,2-xylose, which are absent in humans. Glycoproteins decorated with such monosaccharides may elicit immune responses. The advantage and disadvantage of the insect expression system are similar to those of plant cells. It is also a high-yielding expression system and easy to use, but can incorporate non-human glycan structures, including the α-1,3-fucose moiety, into target glycoproteins (Geisler et al., 2015). The major difference between these two non-mammalian expression systems is that they produce different glycan structures (Figure 5).

The methylotrophic yeast *Pichia pastoris* has also been developed for protein glycoengineering (Cereghino and Cregg, 2000; Bretthauer, 2003; Choi et al., 2008). Compared with mammalian cells, yeast can be cultured at a higher cell density, which makes glycoprotein production more efficient and production costs much cheaper. However, O-linked glycans in *Pichia pastoris* are typically linear chains of oligomannoses and N-linked glycans are of the high-mannose type (Figure 5). Therapeutic glycoproteins carrying such glycan structures can be easily cleared from the body due to the lack of terminal Neu5Ac residues. Recently, bacteria have also attracted great interest in their potential use for protein glycoengineering as a fast, simple, and low-cost expression system (Baker et al., 2013; Merritt et al., 2013; Yates et al., 2018). However, glycans in bacteria are also significantly different from human glycans (Figure 5) (Du et al., 2019; Harding and Feldman, 2019). In order to circumvent the risk of immunogenic reactions from non-human glycans, several approaches to humanizing yeast and bacterial N-glycosylation pathways have been attempted over the last twenty years (Hamilton and Gerngross, 2007). For example, Hamilton et al. (2006) engineered the protein glycosylation pathway in *Pichia pastoris* by knocking out four yeast-specific glycosylation genes and introducing 14 heterologous glycosylation genes. Using humanzied *Pichia pastoris* expression system, they were able to produce hEPO containing predominantly human N-glycans that had greater than 90% terminal sialylation. However, although today there are many selections of non-mammalian systems available for protein glycoengineering, they have not been widely applied in the production of therapeutic glycoproteins, largely due to the complexity of these expression systems.

Non-mammalian cells can also be applied for the glycoengineering of industrial enzymes. Unlike therapeutic proteins, where immune response is a concern, industrial enzymes are not products for direct human use and, therefore, there is no need to humanize the glycosylation pathways in these cells. Currently, the methods for industrial enzyme glycoengineering mainly include changing the structures of glycans on industrial enzymes by switching their expression systems, by optimizing the culture conditions, or by changing the number of glycosylation sites through amino acid mutations. In this research area, one of the relatively more explored industrial enzyme families is the cellulase family (Beckham et al., 2012; Greene et al., 2015). Cellulases are glycoside hydrolases (GHs) that can decompose cellulose in wood, agricultural residues and municipal solid wastes into shorter-chain sugars, such as cellodextrin, cellobiose, and glucose, which could then be converted to bioethanol through a fermentation process. In the process of bioethanol production, the enzymatic activity of cellulase plays a crucial role. In order to improve the performance of cellulase, Adney et al. (2009) inactivated the N-glycosylation site at the position 384 of the *Trichoderma reesei* Family 7 cellobiohydrolase (*Tr*Cel7A) by mutation and expressed the resulting mutant enzyme in a different host, *Aspergillus niger var. Niger*, which is a fungus and one of the most common species of the genus *Aspergillus*. By comparing the bacterial cellulose hydrolysis time courses for the wild-type *Tr*Cel7A and the mutant, they found that the removal of a glycan at N384 resulted in the improvement of the activity of the enzyme by 70% after 120 h. However, although enzyme glycoengineering has received more and more attention in recent years, the glycoengineering outcomes are still not satisfactory. In order to get better results faster, more detailed research is needed to answer some fundamental questions that have not been answered. These questions are essentially the same as the ones for therapeutic protein glycoengineering research: what, how, and why specific glycosylation patterns can improve the performance of enzymes.

## Chemistry-Based Protein Glycoengineering

Over the past 40 years, substantial progress has been made in all aspects of cell-based protein glycoengineering, including optimization of fermentation conditions, genetic modification of glycoprotein expression hosts, glycoprotein purification, composition analysis, and characterization. However, challenges that limit the wide application of this approach in industry and medicine still exist. As aforementioned, the challenges are mainly related to the inseparable and unpredictable nature of glycoform mixtures produced by different cells. Because it is still difficult to precisely and effectively quantify heterogeneous glycoform mixtures, it is not trivial to obtain definitive and reliable information about the changes in properties caused by protein glycoengeering. In order to meet these challenges, scientists have explored various technologies to simplify the complexity of glycoprotein samples, such as those involved the use of protein glycosylation pathway engineering and those based on the use of biochemistry and organic chemistry. These technologies do not in any simple sense replace or exclude each other, but rather complement and enrich each other.

Compared with cell-based technologies, chemistry has the advantage of being relatively more precise and flexible for the production of homogeneous glycoforms of proteins, but has the disadvantage of being more labor-intensive and less useful in large-scale production. In theory, chemistry allows for the small-scale preparation of homogeneous glycoforms with any glycans or any amino acid sequences, which can meet the requirement of structural diversity and representativeness of research samples for both basic research and protein glycoengineering studies. However, in addition to the above-mentioned disadvantage, chemistry as a tool is currently still immature: many crucial steps for glycoprotein synthesis have not been well optimized and most essential starting materials are not commercially available. It is thus still difficult for non-professionals to use chemistry to perform protein glycoengineering.

### Glycoengineering Based on Biochemistry

Protein glycoengineering based on biochemistry methods is mainly accomplished through the use of biochemical reactions catalyzed by a variety of glycosidases and glycosyltransferases (Rothman et al., 1989; Nemansky et al., 1995; Hodoniczky et al., 2005). Glycosidases catalyze the cleavage of glycosidic bonds, while glycosyltransferases catalyze the opposite reaction, glycosidic bond formation, mainly using sugar nucleotides as glycosyl donors. Glycosidases are broadly classified as exo- and endo-glycosidases. Exo-glycosidases sequentially remove monosaccharides from the non-reducing end of glycans. Endo-glycosidases are capable of cleaving specific glycosidic bonds inside the glycan chains.

In recent years, the application of glycosidases and glycosyltransferases to protein glycoengineering, including the development of a cell-free glycoprotein synthesis technology, has greatly advanced this field (Jaroentomeechai et al., 2018; Wen et al., 2018; Kightlinger et al., 2019; Moremen and Haltiwanger, 2019; Rahfeld and Withers, 2020). A prominent aspect in the advance is to make us realize the importance of subtle variations in glycan structures to protein performance (Washburn et al., 2015). A representative example is that by changing the glycosidic linkages between the terminal sialic acid residue and the penultimate galactose residue, Anthony *et al*. was able to greatly improve the therapeutic efficacy of intravenous immunoglobulin (IVIG) (Anthony et al., 2008). As a blood product, IVIG is a treatment for autoimmune diseases including immune thrombocytopenia, rheumatoid arthritis, and systemic lupus erythematosus. Just like many other glycosylated antibodies, its N-linked glycans at amino acid position 297 have many different structures, some without the terminal Nue5Ac and some with α-2,6-linked or α-2,3-linked Neu5Ac (Kaneko et al., 2006). In their work, Anthony et al. (2008) found that when IVIG was treated with α-2,6-neuraminidase, the anti-inflammatory activity of IVIG was completely lost. When digested with α-2,3-neuraminidase, its activity was not affected. This observation suggested that the anti-inflammatory activity of IVIG may be directly correlated with the presence of α-2,6-Neu5Ac. Under the guidance of this hypothesis, they first removed Neu5Ac residues from glycans at the Asn297 site of IVIG-derived Fc fragments with α-2,3/6-neuraminidase, and then use β-1,4-galactosyltransferase and α-2,6-sialyltransferase to increase their homogeneity and α-2,6-sialylation level (Figure 3). Biological tests confirmed that the resulting Fc fragments had the same anti-inflammatory activity at significantly reduced doses. The success of this glycoengineering effort illustrated the importance of increasing the level of glycoprotein homogeneity to enhance the capability of protein glycoengineering.

**Figure 6:**
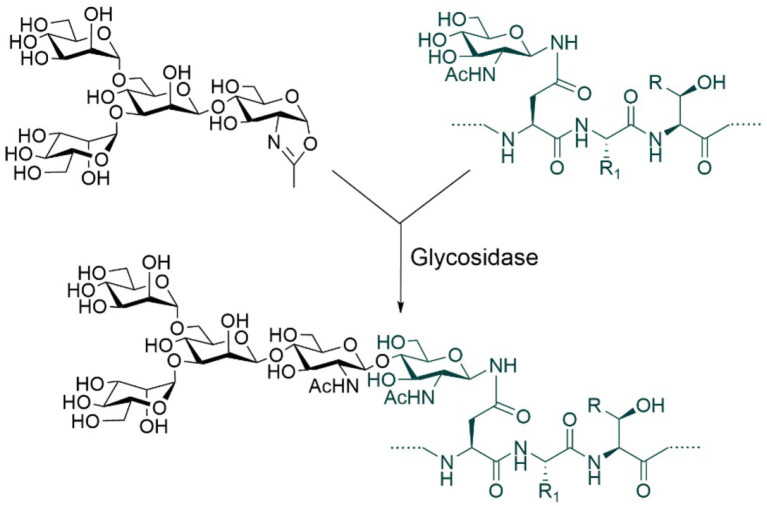
Glycosynthase-catalyzed N-glycosylation using oxazoline donors. R = H or Me, R_1_ = Amino acid side chains.

Although relatively homogeneous glycoproteins can be prepared through the combined use of glycosidases and glycosyltransferases, this is a rather complex process largely due to the current limitations of glycosyltransferases and of the reactions they catalyze. Glycosyltransferases typically add specific monosaccharides one at a time to specific substrates and to specific sites on these substrates. In addition, many glycosyltransferases and sugar nucleotide donors are either expensive or not commercially available. All these facts render it not very straightforward to apply glycosyltransferase-catalyzed multistep reactions to generate a large number of homogeneous glycoforms bearing structurally closely related glycans to meet the research needs of protein glycoengineering. To overcome these limitations, it is necessary to replace the stepwise enzymatic approach with a highly convergent one. The key to achieving a convergent synthesis is to find enzymes that can catalyze the attachment of oligosaccharides relatively non-specifically to a variety of substrates. To meet this demand, a new class of enzymes has been developed. They are named “glycosynthases” (Mackenzie et al., 1998; Malet and Planas, 1998). Glycosynthases are generally derived from glycosidases through genetic mutations. In the presence of activated oligosaccharide donors, glycosynthases can transfer *en bloc* the oligosaccharides onto different glycoprotein acceptors in high yields (Figure 6).

The glycoengineering method based on glycosynthase-catalyzed transglycosylation is similar to that based on glycosyltransferase-catalyzed reactions. It also requires the removal of a large portion of N-linked glycans from glycoproteins by glycosidases before the transglycosylation reaction. This method was invented more than two decades ago and the current most commonly used one employs oligosaccharide oxazolines as donor substrates (Figure 6; Wei et al., 2008; Wang et al., 2019). The development of glycosynthase enzymes has helped solve the problem of structural diversity of glycoforms required by glycoengineering research to a certain extent. For example, Lin et al. (2015) were able to generate more than a dozen of homogeneous antibody glyco-variants, i.e., variants with different glycosylation patterns, using this type of enzymes. By comparing the activities of synthetic glyco-variants, they found that the complex-type biantennary N-glycans with two terminal α-2,6-linked Neu5Ac residues seemed to be optimal structures. Antibodies modified with such glycans showed enhanced activities against cancer, influenza, and inflammation.

In addition to increasing the diversity of glycoforms for research, the glycosynthase-based method can also be applied to achieve glycosylation site selectivity, that is, attaching different glycans to different glycosylation sites. In an example of such study, Giddens et al. (2018) successfully prepared several antibody variants with different N-glycans at the glycosylation sites in their Fc and Fab regions through the combined use of three endoglycosidases (Endo-S, Endo-S2, and Endo-F3), 1,6-fucosidase from *Lactobacillus casei*, and endoglycosidase mutants. They found that the antibody containing sialylated N-glycans on the Fab fragments and non-fucosylated ones on the Fc fragments had enhanced binding capacity to the FcγRIIIa receptor and greatly improved ADCC activity.

The advantage of *in vitro* enzymatic glycoengineering is that, by improving the structural control of protein glycosylation, it allows for relatively easy access to homogeneous glycoforms. With such research samples, quantitative structure-function relationships can be derived to guide the design of new protein glycoengineering research. However, the efficiency of this method is currently still limited due to the limited commercial availability of oligosaccharide substrates, limited range of substrates that can be tolerated by glycosynthase enzymes and the difficult-to-control glycosylation site selectivity. It is also challenging to use this method in large-scale. Because of these limitations, the diversity and quantity of generated samples may not be high enough to meet the requirement, and thus the research process could be slow and the identified glycoforms may not be the best choices for future use. In addition, because of the differences in the enzymes involved in protein O-glycosylation, it is currently still difficult to enzymatically transfer oligosaccharides *en bloc* to O-glycosylation sites. But thanks to the development of useful software like ISOGlyP and NetOglyc, it is now possible to predict O-glycosylation sites based on sequence and structure features of proteins (Hansen et al., 1998; Leung et al., 2014).

### Glycoengineering Based on Organic Chemistry

Engineering O-linked protein glycosylation can be achieved by organic synthesis. This technique can also significantly expand the structural diversity of glycoforms. These advantages mainly come from the more precise and flexible nature of organic synthesis. Unlike many other methods, organic synthesis enables the modification of glycoprotein structures at the atomic level. In theory, it could allow scientists to prepare glycoforms containing any number of glycosylation sites and any type of glycan structures that are required for protein glycoengineering research (Price et al., 2012; Chaffey et al., 2018).

In the past two decades, with the development of synthetic methods for the preparation of glycans and proteins, total chemical synthesis of glycoproteins was also developed (Fernandez-Tejada et al., 2015). The current strategy for glycoprotein synthesis relies on native chemical ligation/metal-free desulfurization (NCL/MFD) to connect synthetic peptides and glycopeptide fragments together. After that, the resulting long glycopeptide chains can be folded *in vitro* to form biologically active glycoproteins. The peptides for glycoprotein synthesis can be prepared from commercially-available protected amino acids by solid-phase peptide synthesis (SPPS). N-glycopeptides can be synthesized by condensation of glycosyl amines with side-chain-unprotected aspartic acids in partially protected peptides. O-linked glycopeptides can be made by incorporating O-glycosylated amino acid building blocks during SPPS.

Among the studies undertaken, the most representative one is the case of the chemical glycoengineering of hEPO. In their study, Wang et al. (2013) first applied the NCL/MFD technology to assemble the sequence of glycosylated hEPO from three N-glycopeptides, one O-glycopeptide and one peptide, which was then folded in a cysteine-cystine redox system to produce the final three-dimensional structure of hEPO. The resulting glycoform has the expected biological activity (Figure 4). This work for the first time provided sufficient experimental evidence for the feasibility of protein glycoengineering based on a chemical synthesis strategy, and laid a solid foundation for further development in this research area.

Using chemical approaches, two research groups were able to develop new guidelines for N- and O-linked glycoengineering of proteins (Chaffey et al., 2018). In their work, Price et al. (2012) provided a theoretical principle to guide the design of protein N-glycoengineering, which stated that “incorporating the enhanced aromatic sequons into appropriate reverse turn types within proteins should enhance the well-known pharmacokinetic benefits of N-glycosylation-based stabilization by lowering the population of protease-susceptible unfolded and aggregation-prone misfolded states”. An enhanced aromatic sequon normally is a five- or six-residue sequence that contains an aromatic amino acid being located two or three resides away from the N-terminus of the consensus sequence of N-linked glycosylation (Asn-Xaa-Thr/Ser). The five-residue sequence forms a type I β-turn, while the six-residue one forms a type II β-turn. This principle was confirmed in practical applications like the N-glycoengineering of β-sheet-rich 34-residue WW domain from the human Pin1 protein (Pin WW). By replacing the loop 1 of Pin WW with a five-residue enhanced aromatic sequon, Phe16-Ala18-Asn19-Gly20-Thr21, and glycosylating the Asn19 with N-GlcNAc, Price et al. (2011) were able to significantly increase its melting temperature.

By systematically studying the effects of O-linked glycans on the properties of a family 1 carbohydrate binding module, Patrick et al. established a guideline for protein O-glycoengineering, which stated that “O-linked glycoforms with better overall properties can be generated by collaboratively varying glycan structures and adjacent amino acids within unstructured regions that are important for biological function and/or susceptible to proteolytic cleavage and other undesired degradation reactions” (Chaffey et al., 2018). The validity of this guideline was confirmed by the glycoengineering study of a therapeutic protein, human insulin. In this study, they demonstrated that O-mannosylation of insulin B-chain Thr27 reduced its susceptibility to proteases and self-association (Guan et al., 2018).

However, although protein glycoengineering based on organic chemistry has some advantages, it also has a big disadvantage, that is, organic synthesis of glycoproteins as a new technology has not been well optimized and currently, it can only be utilized by experienced researchers. In order to make this glycoengineering approach widely accepted and used, more efforts need to be put to improve the synthesis of oligosaccharides and glycopeptides and the efficiency of the ligation of peptide/glycopeptide fragments. Perhaps more importantly, it is necessary to expedite the commercialization process of glycan building blocks, oligosaccharides, and glycopeptides and even synthetic glycoforms, because the easy access to these substances usually could help scientists gain and maintain their interest in a research area.

## Conclusions and Outlook

Protein glycoengineering as an important way to improve the performance of therapeutic proteins and industrial enzymes has attracted substantial interest over the past few decades (Neustroev et al., 1993; Elliott et al., 2003). However, due to the lack of reliable guidance, this technology is still in its infancy, and the degree of its acceptance in the scientific community is not high. At present, because biology-based methods are relatively easy to implement, some of them, especially those involving the manipulation of protein N-glycosylation pathway are more frequently employed in protein engineering research. Although such methods provide some results more quickly, the results may have some uncertainty due to the heterogeneity and low purity of research samples (Mimura et al., 2018). Chemistry-based methods, especially organic synthesis, can help overcome some of the uncertainty issue because they can produce structurally defined homogeneous glycoforms. But organic synthesis has its own weakness. It is difficult to use and is complex, expensive and time-consuming.

In order to solve the present predicament, these different methods need to be better combined to increase the practical applicability and the success rate of protein glycoengineering. A possible combination strategy is as follows: organic and/or enzymatic synthesis is used to deeply understand the structure-property relationships of representative model glycoproteins that have relatively small sizes and simple structures. Theoretical predictions derived from the high-level understanding of protein glycosylation can then be used to guide protein glycoengineering efforts (Umana and Bailey, 1997; Grainger and James, 2013; Spahn et al., 2016, 2017; Krambeck et al., 2017; Sokolov et al., 2018; Liang et al., 2020). Finally, cell-based methods can be used to more quickly obtain designed glycoforms in large-scale. Previous studies have suggested the feasibility of this strategy. It is expected that such a strategy, once fully established, should greatly promote the advancement of protein glycoengineering in the future.

## Author Contributions

BM, XG, YL, SS, JL, and ZT wrote the paper. All authors have given approval to the final version of the manuscript.

## Conflict of Interest

The authors declare that the research was conducted in the absence of any commercial or financial relationships that could be construed as a potential conflict of interest.
